# High correlation between skin color based on CIELAB color space, epidermal melanocyte ratio, and melanocyte melanin content

**DOI:** 10.7717/peerj.4815

**Published:** 2018-05-24

**Authors:** Wen-Shyan Huang, Yi-Wen Wang, Kun-Che Hung, Pai-Shan Hsieh, Keng-Yen Fu, Lien-Guo Dai, Nien-Hsien Liou, Kuo-Hsing Ma, Jiang-Chuan Liu, Niann-Tzyy Dai

**Affiliations:** 1 Division of Plastic and Reconstructive Surgery, Zuoying Branch of Kaohsiung Armed Forces General Hospital, Kaohsiung, Taiwan, R.O.C.; 2 Department of Biology and Anatomy, National Defense Medical Center, Taipei, Taiwan, R.O.C.; 3 Division of Plastic and Reconstructive Surgery, Department of Surgery, Tri-Service General Hospital, National Defense Medical Center, Taipei, Taiwan, R.O.C.; 4 Department of Orthopedics, Shuang Ho Hospital, Taipei Medical University, Taipei, Taiwan, R.O.C.

**Keywords:** Hypopigmentation, Color reader, CIELAB color space, Skin tissue engineering, Pigment cell

## Abstract

**Background:**

To treat skin color disorders, such as vitiligo or burns, melanocytes are transplanted for tissue regeneration. However, melanocyte distribution in the human body varies with age and location, making it difficult to select the optimal donor skin to achieve a desired color match. Determining the correlations with the desired skin color measurement based on CIELAB color, epidermal melanocyte numbers, and melanin content of individual melanocytes is critical for clinical application.

**Method:**

Fifteen foreskin samples from Asian young adults were analyzed for skin color, melanocyte ratio (melanocyte proportion in the epidermis), and melanin concentration. Furthermore, an equation was developed based on CIELAB color with melanocyte ratio, melanin concentration, and the product of melanocyte ratio and melanin concentration. The equation was validated by seeding different ratios of keratinocytes and melanocytes in tissue-engineered skin substitutes, and the degree of fitness in expected skin color was confirmed.

**Results:**

Linear regression analysis revealed a significant strong negative correlation (*r* =  − 0.847, *R*^2^ = 0.717) between CIELAB L* value and the product of the epidermal melanocyte ratio and cell-based melanin concentration. Furthermore, the results showed that an optimal skin color match was achieved by the formula.

**Discussion:**

We found that L* value was correlated with the value obtained from multiplying the epidermal melanocyte ratio (R) and melanin content (M) and that this correlation was more significant than either L* vs M or L* vs R. This suggests that more accurate prediction of skin color can be achieved by considering both R and M. Therefore, precise skin color match in treating vitiligo or burn patients would be potentially achievable based on extensive collection of skin data from people of Asian descent.

## Introduction

Evaluating skin color match for treating skin color disorder requires an objective measurement to quantify visual skin color into numerous levels. The principles of color measurement established by the Commission International d’Eclairage (CIE) have been widely applied to skin. Color values have been obtained by using reflectance spectroscopy, expressed in terms of color space *L*^∗^ value, hue angle, and chroma values (Weatherall & Coombs, 1992). Differences of trichromatic color vision between individuals are identified in terms of one, two, or all three CIE color-space parameters: *L*^∗^ value, *a*^∗^ value, and *b*^∗^ value (CIELAB). The *L*^∗^ value, which correlates to perceived lightness and ranges from absolute black (0) to absolute white (+100), is the most sensitive of the trichromatic values to skin color change. Because the method is quantitative and the principles are internationally recognized, these color-space parameters are proposed for the unambiguous communication of skin color information that relates directly to visual observations of clinical importance or scientific interest (Stevenson et al., 2012).

Medical therapy for hypopigmentation disorders has improved in recent years; however, complete repigmentation and perfect skin color match seem to be unsatisfactory in most patients treated (Iannella et al., 2016). Therefore, a variety of surgical grafting techniques have been performed to treat skin color disorders that do not respond to medical treatment, such as split thickness grafts, cultured autologous melanocytes, minigrafts, suction blister grafts, and non-cultured epidermal suspension, among others (Gauthier & Benzekri, 2012). These techniques contain different advantages and disadvantages with respect to cost, time consumption, treatment area and location, need for equipment, and possible outcome of abnormal appearance (Gauthier & Benzekri, 2012). A previous study reported that cultured skin substitutes (CSS) fabricated from autologous keratinocytes and fibroblasts seeded onto collagen-glycosaminoglycan substrates could be applied to excised, full-thickness burns on five patients. Spontaneous repigmentation of CSS treatment from passenger melanocytes in keratinocyte culture was found within 2 months after grafting (Harriger et al., 1995). Kahn & Cohen (1995) used very thin epithelial sheet grafts harvested by dermatome from pigmented donor areas and then covered it with petrolatum gauze on five patients with stable vitiligo, which all resulted in excellent repigmentation and no scarring developed. Gupta, Shroff & Gupta (1999) collected the donor epidermal sheets from the blisters developed through the cutaneous suction apparatus, and then grafted onto the denuded skin lesion site. Alternatively, Falabella (2001) implanted very small dermo-epidermal grafts on recipient sites prepared with minipunches of similar size. Mulekar (2003) and Van Geel et al. (2001) both grafted non-cultured melanocyte-keratinocyte suspensions onto previously dermabraded vitiligo lesions and achieved a high repigmentation. Transplantation of pure primary melanocyte cultures has also been proposed with a better response, relatively homogeneous skin color, and capability for use on larger lesion areas (Chen et al., 2004; Kaufmann et al., 1998; Lontz et al., 1994; Olsson & Juhlin, 2002).

Currently, the outcomes of repigmentation through pigment skin tissue engineering or pigment cell transfer are still unpredictable. Previous studies indicated that regulating cutaneous pigmentation in cultured skin substitutes was feasible by titration of human melanocytes and keratinocytes (Swope, Supp & Boyce, 2002; Swope et al., 1997). However, epithelial melanocytes used in Swope’s experiments came from a single donation; thus, the influence of different sources on skin pigmentation could not be determined. The aim of this work was to identify how melanocyte numbers and activity from different Asian people modulate skin color via a well-defined correlation between skin color and melanocytes. A total of 15 human adult foreskin samples donated from Asian individuals were analyzed and the correlations among skin color determined by CIELAB color-space parameter: *L*^∗^ value, epidermal melanocyte ratio, and melanin content per 10^6^ melanocytes was investigated. Finally, we evaluated the feasibility of applying this skin color relationship in skin tissue engineering.

## Materials and Methods

### Materials

Gibco Company (New York, NY, USA) supplied Trypsin-EDTA solution (10 ×) and Penicillin-Streptomycin (10,000 units/ml penicillin G sodium, 10,000 µg/ml streptomycin sulfate in 0.85% saline). Melanocyte culture medium contains Medium 254 (Cascade Biologics Inc., Portland, OR, Portland), 1% Human Melanocyte Growth Supplement (HMGS, Cascade Biologics Inc., Portland, OR, Portland), and 1% Penicillin-Streptomycin. The EpiLife^®^ keratinocyte medium (contain 0.06 mM calcium chloride) was obtained from Cascade Biologics Inc. (Portland, OR, Portland). Poly (ε-caprolactone) (PCL, CAPA 6500, MW 50,000 g/mol) was purchased from Solvay (Warrington, UK). Type I collagen from calfskin was purchased from Sigma (St. Louis, MO, USA). Dichloromethane (DCM) was purchased from J. T. Baker (Phillipsburg, NJ, USA).

### Inclusion criteria for skin samples collection

A total of 15 Asian young adult foreskin samples were collected during circumcision surgery in Tri-Service General Hospital, R.O.C. Mean age of patients was 24.47 ± 1.03 years old, ranging from 21 to 38 years. The study protocol was reviewed and approved by the Institutional Review Board (IRB) in the Tri-Service General Hospital, R.O.C. (TSGHIRB No.: 095-05-0068). Written informed consent was obtained from each donor.

### Primary culture of human epidermal keratinocytes and melanocytes

Primary human epidermal keratinocytes (PHEKs) and melanocytes (PHEMs) were isolated from equal size (1 cm × 1 cm in square) of human young adult foreskin samples obtained in the surgery of circumcision. For culture of PHEKs, the foreskin sample was initially immersed in 10 ml of 0.2% Dispase II solution (Sigma, St. Louis, MO, USA) at 4 °C for 48 h and diced in pieces, followed by incubation in 0.05% Trypsin-EDTA solution for 15 min. The pelleted cells were obtained by a centrifugation at 1,300 rpm for 5 min, seeded in a fibronectin/collagen (AthenaES, Baltimore, MD, USA) coated flask and cultured in EpiLife^®^ keratinocyte medium at 37 °C in 5% CO_2_. For primary culture of PHEMs, the epidermal cell cultured in the EpiLife^®^ medium were transferred to melanocyte culture medium after the epidermal primary culture and incubated at 37 °C in 5% CO_2_. Highly selected culture of PHEMs was then obtained after passage 2.

### Skin color measurement

Foreskin samples were obtained immediately after surgery, blood and adipose tissues were removed, and color was measured in triplicate for each sample *ex vivo* using a color reader CR-10 (Konica Minolta, Osaka, Japan). The color differences were displayed in terms of trichromatic *L*^∗^, *a*^∗^, and *b*^∗^ values as determined by the CIE. Since the *L*^∗^ value correlates to perceived color brightness (black vs. white) and is the most sensitive trichromatic value for measuring skin pigmentation in the pilot study, it was chosen rather than *a*^∗^ and *b*^∗^ values to represent measured skin color in this study. The *L*^∗^ value was measured in triplicate by detecting the top surface of the collected foreskin sample and recorded as mean ± SE (standard error).

### Analysis of melanocyte ratio in epidermal cells

Fluorescent-activated cell sorting (FACS) was used to distinguish melanocytes from epidermal cells. Epidermal cell suspensions of foreskin were immediately prepared after surgery, which were then centrifuged at 300 × g for 5 min and the cell pellets were treated with a Cytofix/CytoPerm Plus kit (BD, Franklin Lakes, NJ, USA) for subsequent flow cytometry. Briefly, melanocytes were isolated from epidermal cells by adherence. Next, the cells were permeabilized with 200 µl Cytofix/CytoPerm solution for 20 min at 4 °C and washed with 1 ml Perm/Wash Buffer (BD, Franklin Lakes, NJ, USA) twice. For labeling melanocytes, permeabilized cells were stained with mouse anti-human melan-A IgG (Santa Cruz Biotechnology, CA, USA) and incubated with fluorescein isothiocyanate-conjugated (FITC) goat anti-mouse IgG (Jackson, PA, USA). Negative controls for melan-A staining consisted of cells stained with FITC goat anti-mouse IgG only. The samples were then analyzed with five replicates being used for each sample, the samples were analyzed on a FACS Calibur flow cytometer using CellQuest software (BD, Franklin Lakes, NJ, USA); the mean value was then obtained.

### Determination of melanin concentration

The melanin production of melanocytes from distinct foreskin samples in term of total melanin content per 10^6^ melanocytes was measured by a spectrophotometric assay. Briefly, Purified primary human epidermal melanocytes (PHEMs) (10^6^ cells per pellet) were lysed with 1 M NaOH at 80 °C for 2 h. After centrifugation at 12, 000 × g for 10 min at room temperature, the supernatants were transferred to fresh tubes and melanin content was determined in triplicate for each sample by measuring the absorbance at 490 nm in a spectrophotometer and expressed as microgram of melanin per 10^6^ cells. Synthetic melanin (Sigma, St. Louis, MO, USA) was used to plot a standard curve.

### Preparation of collagen/PCL membranous scaffolds

Collagen/polycaprolactone (PCL) scaffolds were prepared as described previously (Dai et al., 2004). Briefly, type I collagen was dissolved in 1% acetic acid, generating a 0.25% w/v collagen solution. The collagen solution was poured into a round glass vial (diameter 2.5 cm and height 4.5 cm) and frozen at −20 °C for 50 min, followed by lyophilizing in a freeze-dryer (DRC-1100, Eyela, Japan) for 24 h. The PCL/dichloromethane (DCM) solution (2.5% w/v) was then added to the freeze-dried collagen matrix to prepare a 1:20 w/w collagen/PCL scaffold. The glass vial was kept open overnight to allow DCM evaporation.

### Color measurement of pigmented tissue-engineered skin substitutes based on collagen/PCL scaffold

Pigmented tissue engineered skin substitutes (diameter 2.5 cm) with primary human epidermal keratinocytes (PHEKs) and/or PHEMs (total cell density of 4.72 ×10^4^ cells/cm^2^) in various ratios were prepared based on the *L*^∗^ value of calculated skin color relationship including L40 (melanocyte ratio was 1.79), and L50 (melanocyte ratio was 4.83) (*L*^∗^ values as 40 and 50 respectively) groups. Epilife serum-free medium was used to incubate the skin substitute at 37 °C in 5% CO_2_. The 6-well tissue culture plastics without cells served as the blank group. The color of pigmented tissue-engineered skin substitutes was measured with a color reader at various time points (10, 20, 30, 40, 50, 60, and 70 days).

### The analysis of correlation for skin color and melanocytes

To estimate the relationships of epidermal melanocyte ratio or melanin concentration vs skin lightness based on foreskin CIELAB *L*^∗^ value, linear regression analysis was performed. The coefficient of determination (*R*^2^) and Pearson’s correlation coefficient (*r*) were calculated to measure goodness of fit of a statistical model and the strength and direction of the linear relationship.

### Statistical analysis

The continuous data such as *L*^∗^ value of foreskin samples and tissue-engineered skin substitutes, melanin amount, and the melanocyte ratio in epidermal cells were shown as “mean  ± SD (SD: standard deviation)”. The variables were grouped first, comparing mean values in categories, Pearson’s correlation coefficient (r) and coefficient of determination consecutively. Simple linear regression analysis was used afterwards to evaluate the combined effect of several variables and to estimate the coefficient of determination. Statistical Product and Service Solutions (SPSS) was used to perform a stepwise forward selection procedure. Thus, for each iterative loop of this procedure one more variable was integrated into the new formula. All statistical result is statistically significant when the *P* value is less than 0.05 (*P* < 0.05). Statistical analysis was performed using Statistical Package for the Social Sciences, Version 12.0 (SPSS Inc., Chicago, IL, USA).

## Results

### Measurement of skin color (*L*^∗^ value)

**Figure 1:**
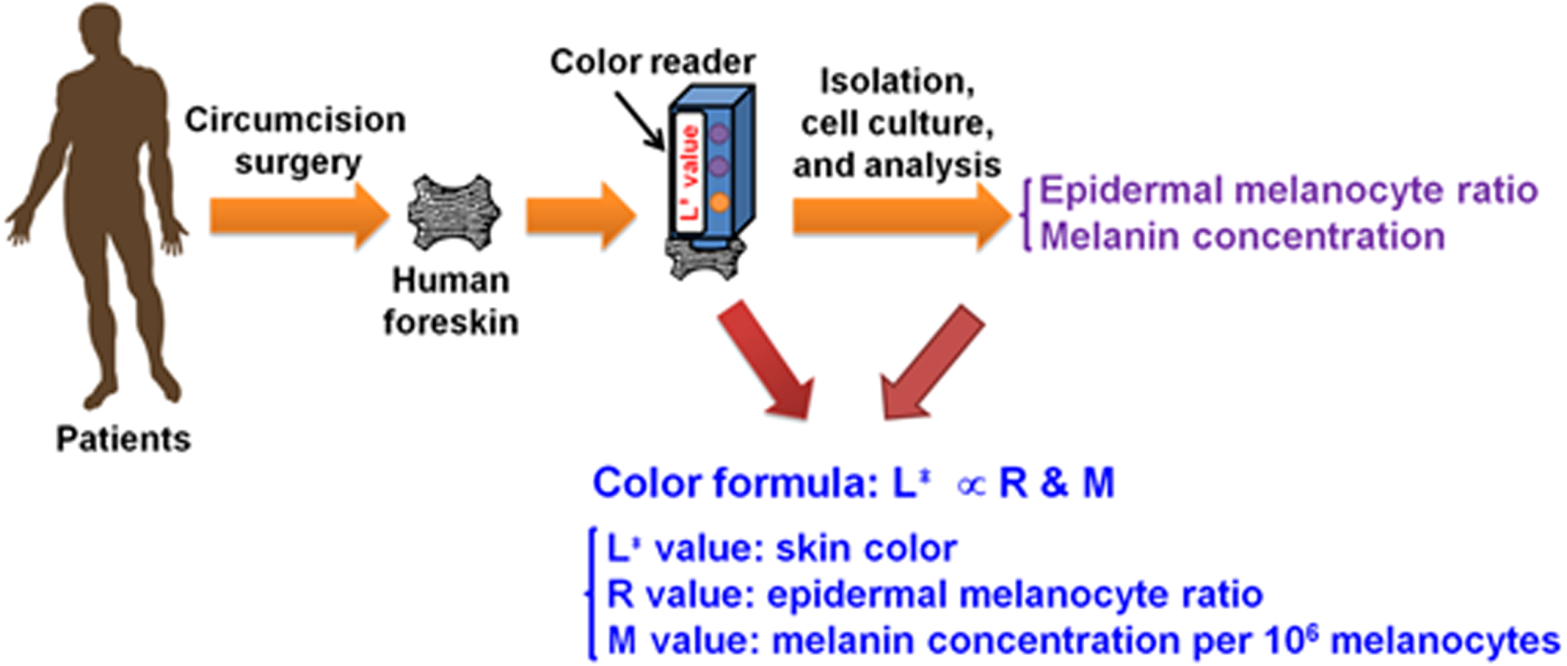
The illustration of the process for measurement of skin color and formulation of the relation among *L*^∗^ value, epidermal melanocyte ratio and melanin concentration.

The study design of measuring skin color of foreskin samples, melanin concentration, and epidermal melanocyte ratios for establishing a relationship with skin color is shown in Fig. 1. The skin color of 15 human young adult foreskin samples was measured immediately after surgery by a color reader *ex vivo* yielding values from 39.43 ± 0.21 to 52.37 ± 1.90 as determined by CIELAB color-space parameter: *L*^∗^ value (Table 1).

**Table 1:** Patient profile of foreskin and demographic data (*n* = 3 for *L*^∗^ value and melanin concentration; *n* = 5 for epidermal melanocyte ratio; values are mean ± SD)^a^.

Samples (No.)	Age (years)	*L*^∗^ value	Epidermal melanocyte ratio (%)	Melanin concentration (µg/10^6^ melanocytes)
1	23	39.43 ± 0.21	1.96 ± 0.09	68.96 ± 0.20
2	25	47.27 ± 1.90	3.18 ± 0.24	34.61 ± 0.39
3	24	41.20 ± 2.17	3.13 ± 0.10	41.50 ± 0.34
4	24	46.97 ± 1.52	1.40 ± 0.28	81.91 ± 1.92
5	24	52.13 ± 1.62	1.97 ± 0.31	25.88 ± 0.11
6	22	52.37 ± 1.90	1.44 ± 0.09	21.97 ± 0.62
7	22	45.93 ± 0.59	2.70 ± 0.10	47.43 ± 0.72
8	26	49.30 ± 0.60	3.49 ± 0.14	24.31 ± 0.58
9	24	44.07 ± 0.15	2.44 ± 0.10	43.32 ± 2.66
10	22	47.53 ± 2.37	2.71 ± 0.52	26.39 ± 0.68
11	21	48.70 ± 2.81	3.40 ± 0.22	26.97 ± 0.27
12	38	49.73 ± 1.15	3.58 ± 0.07	23.39 ± 0.32
13	23	47.87 ± 3.26	3.23 ± 0.22	17.99 ± 0.50
14	25	45.87 ± 2.18	1.68 ± 0.12	67.66 ± 0.46
15	24	50.60 ± 1.40	1.41 ± 0.14	36.07 ± 0.20

**Notes.**

a *n*, the number of tests performed on each individual sample.

### Melanocyte ratio in epidermal cells (R)

The concentration of melanocytes in foreskin epidermal cell suspensions was measured using FACS. The results showed ratios of melanocytes to epidermal cells ranging from 1.40 ± 0.28 to 3.58 ± 0.07% (Table 1).

### Epidermal melanin production (M)

**Figure 2:**
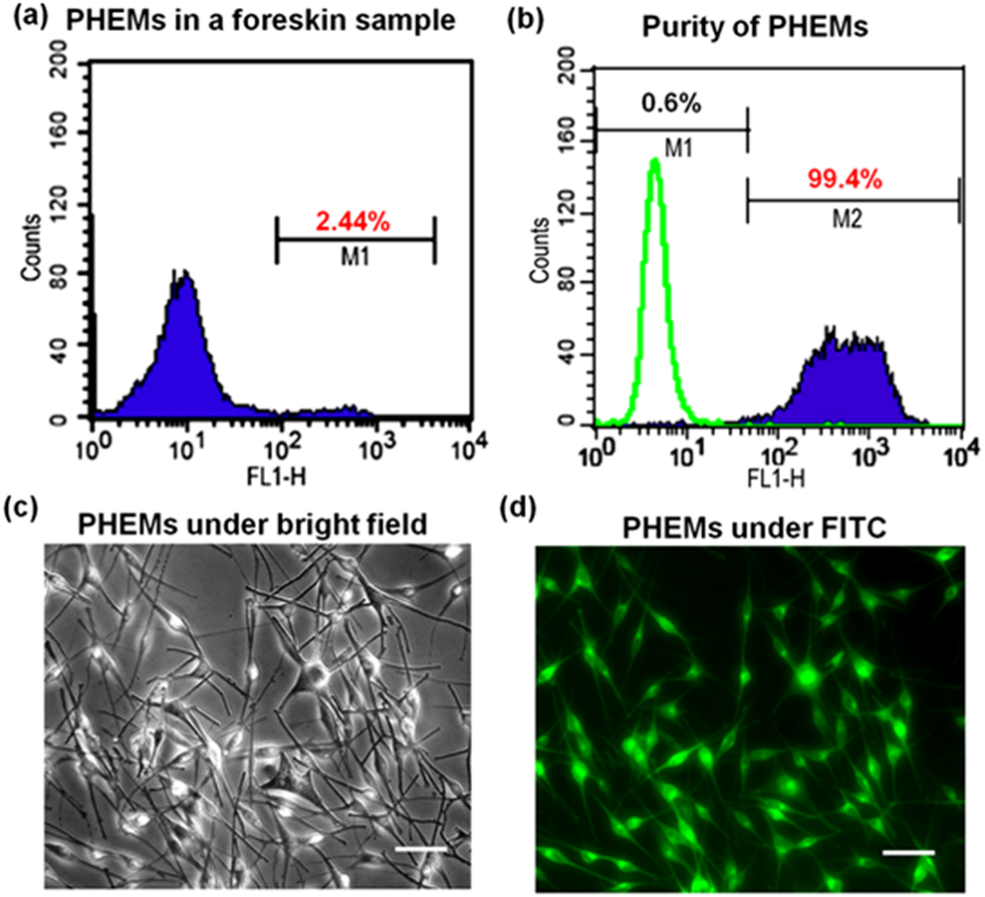
Determination of epidermal melanocyte ratio (100×; scale bar: 100 µm). (A) The foreskin samples were treated immediately with the FACS method. The results showed up to 2.44% of melanocytes (M1) in a human adult foreskin sample. (B) The results of fluorescent-activated cell sorting (FACS) showed up to 99.4% purity of melanocytes (M2) in a selected PHEMs culture. (C) Selected primary culture of human epidermal melanocytes (PHEMs) was shown in bright field. (D) The PHEMs in selected primary culture were labeled by an anti-melan antibody incorporated with fluorescein isothiocyanate (FITC).

To determine the melanin productivity of melanocyte isolated from each foreskin, PHEMs were cultured for two passages reaching a purity of 99.4% and analyzed using immunohistochemistry assays and FACS (Fig. 2). The results showed that the melanin production of 15 PHEMs ranged from 17.99  ± 0.50 to 81.91 ± 1.92μg/10^6^ cells (Table 1).

### The analysis of correlation for skin color

**Figure 3:**
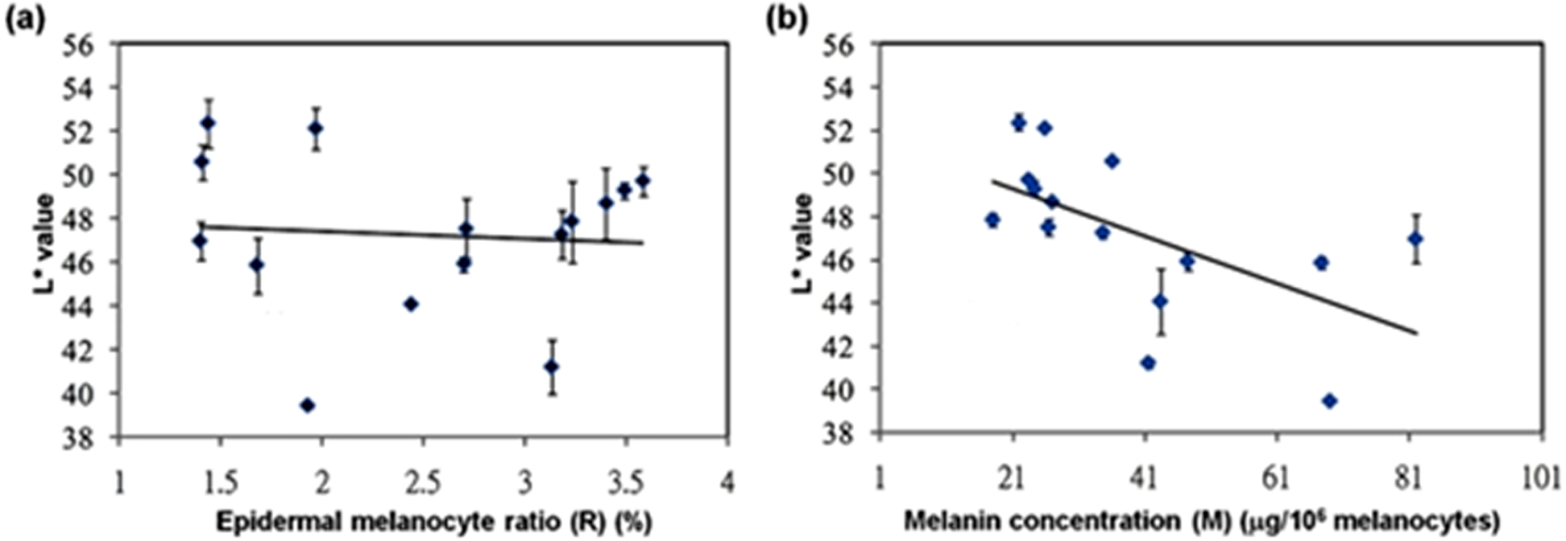
Statistical correlations between skin color-space parameter: *L*^∗^ value and epidermal melanocyte ratio or melanin concentration. (A) *L*^∗^ value corresponding to epidermal melanocyte ratio expresses a random distribution where the relation between them seem to be fairly weak. Correlation coefficient, *r* =  − 0.081 (*P* = 0.774) and coefficient of determination, *R*^2^ = 0.0066. (B) A slight trend of a higher *L*^∗^ value corresponding to a higher melanin concentration per 10^6^ melanocytes was observed. Correlation coefficient, *r* =  − 0.592 (*P* = 0.02) and coefficient of determination, *R*^2^ = 0.3503.

**Figure 4:**
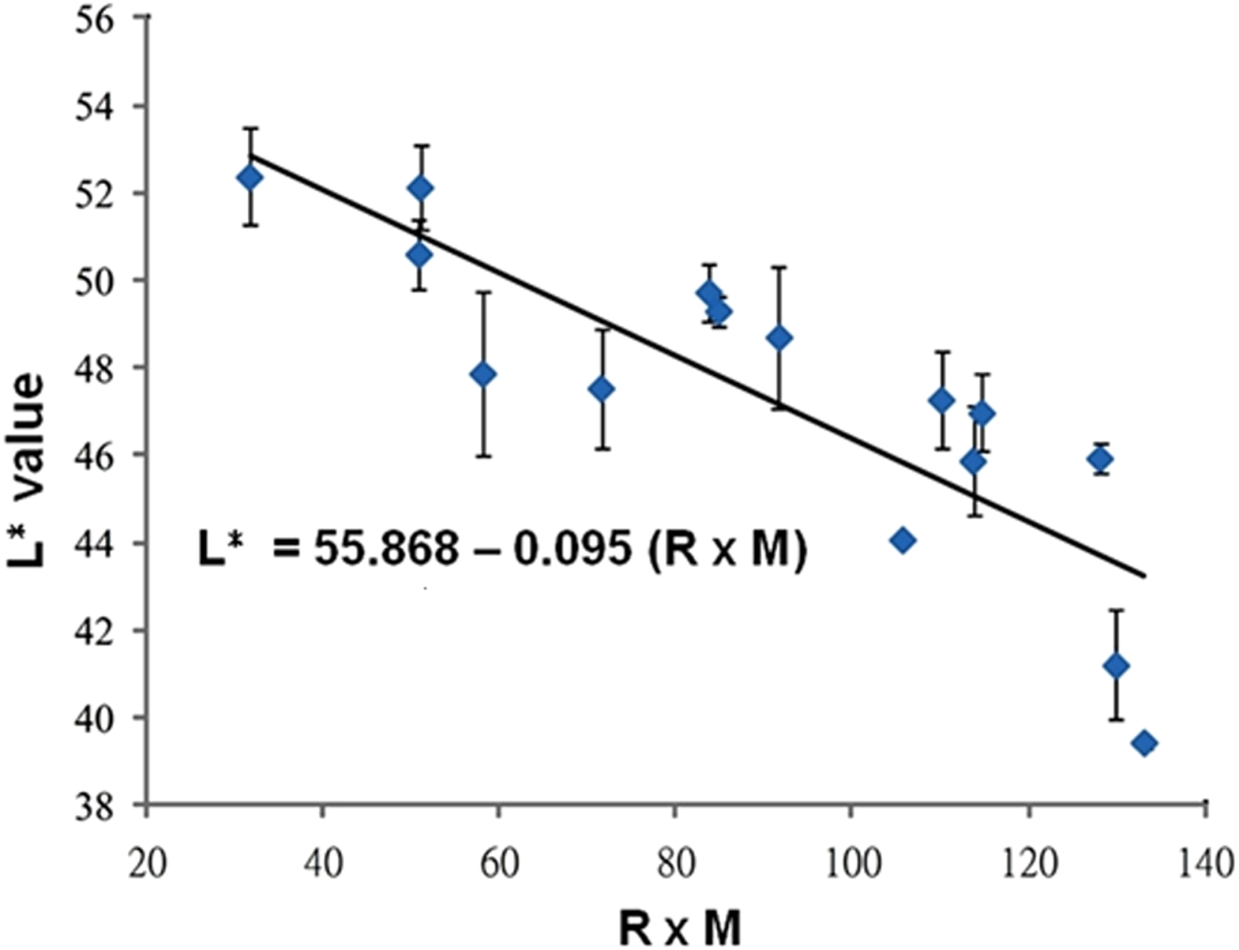
Statistical correlations between skin color-space parameter: *L*^∗^ value and the product of epidermal melanocyte ratio and melanin concentration. A strong correlation was shown between *L*^∗^ value and the value of epidermal melanocyte ratio (R) (%) multiplied by melanin concentration (M) (µg/10^6^ melanocytes). Correlation coefficient, *r* =  − 0.847 (*P* < 0.001) and coefficient of determination, *R*^2^ = 0.717.

We next examined the correlation between skin color and melanin concentration. Plots of *L*^∗^ value against epidermal melanocyte ratio and *L*^∗^ value against melanin concentration per 10^6^ melanocytes were generated based on the data shown in Table 1 (Fig. 3). The results revealed no correlation between *L*^∗^ value and epidermal melanocyte ratio (*r* =  − 0.081, *R*^2^ = 0.0066). However, *L*^∗^ value was negatively correlated with melanin concentration (*r* =  − 0.592, *R*^2^ = 0.3503) (*P* = 0.02) (Fig. 3). However, there were significant associations between the *L*^∗^ value with the production of epidermal melanocyte ratio and melanin content. A further linear regression analysis showed a significantly strong negative correlation (*r* =  − 0.847, *R*^2^ = 0.717) between *L*^∗^ value and the epidermal melanocyte ratio multiplied by cell-based melanin concentration (Fig. 4). Given these results, an equation describing skin color relationship was generated: as *L*^∗^ = *a* × (*M* × *R*) + *b* (*M*: melanin concentration per 10^6^ melanocytes; R: epidermal melanocyte ratio; *a* =  − 0.095; and *b* = 55.872).

### Application of the skin color relationship in skin tissue engineering

**Figure 5:**
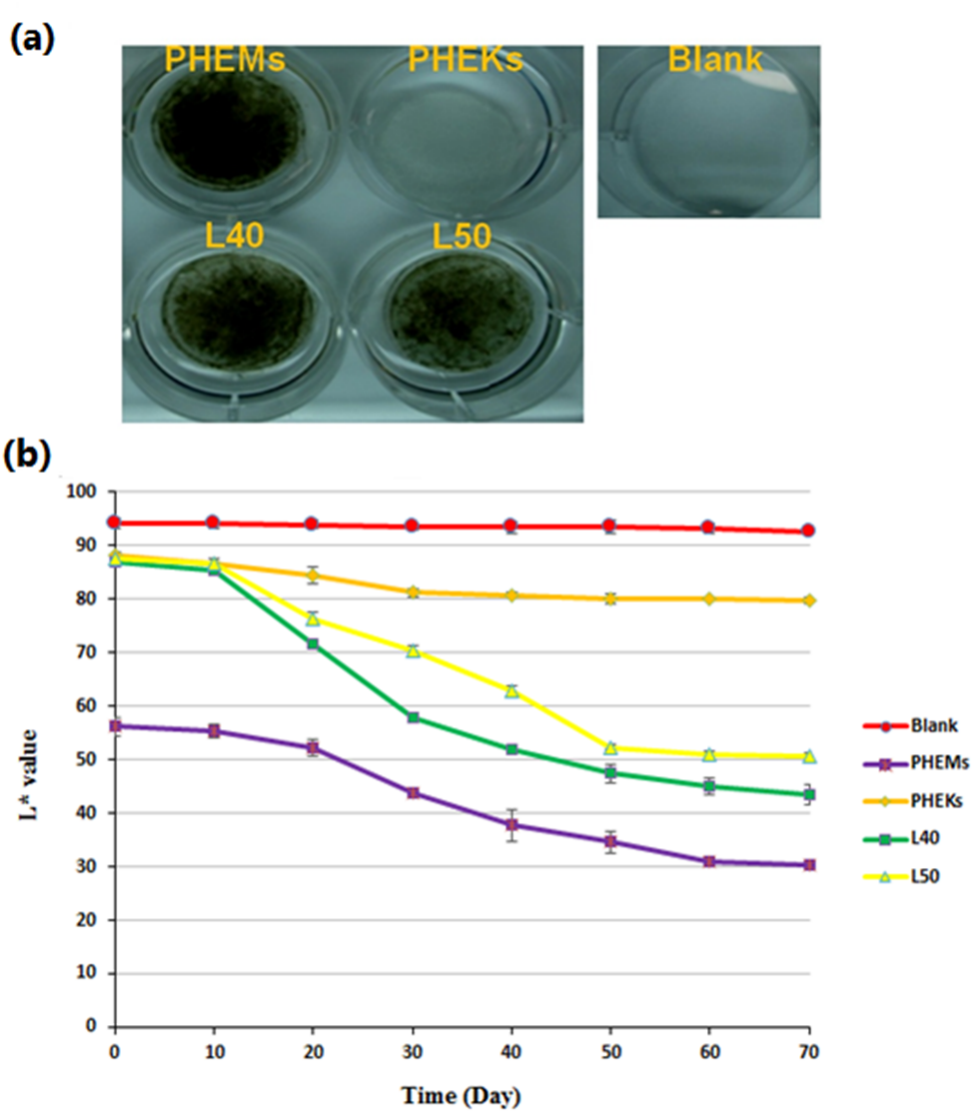
(A) The gross appearance of the collagen/PCL constructs at 70 days; (B) the *L*^∗^ value profiles of the collagen/PCL constructs during a period of 70 days. The 6-well tissue culture plastics without cells served as the blank group.

As shown in Fig. 5A, collagen/PCL constructs at 70 days were ranked in order of different shades of black as PHEMs > L40 > L50 > PHEKs > Blank. The result of the *L*^∗^ value profile of the collagen/PCL constructs is shown in Fig. 5B. Obvious changes of the *L*^∗^ values were noted in 20 days post-culture for all groups except the blank control group. After 70 days, the *L*^∗^ value of L40, and L50 groups were 43.50 ± 1.87, and 50.67 ± 0.55, respectively, whereas PHEMs and PHEKs exhibited values of 30.30 ± 0.56 and 79.73 ± 0.60, respectively.

## Discussion

To date, there is no method to accurately estimate transplanted skin pigments via a skin color formula used to fabricate tissue-engineered skin. Numerous previous studies have shown that skin constitutive pigmentation is determined by melanin production levels. For example, Alaluf et al. (2002b) performed correlation analysis and found the best correlation between the *L*^∗^ value and total melanin content in the epidermis. Del Bino et al. (2015) reported that total melanin content, including eumelanin and pheomelanin content, determines the constitutive skin pigmentation. Wakamatsu et al. (2006) indicated that cell-based melanin production and the predominant biological forms of melanin produced by melanocytes affect skin pigmentation. Additionally, implanting different numbers of melanocytes influences skin color. Swope, Supp & Boyce (2002) implanted 1.1 × 10^2^, 1.1 × 10^3^, and 1.1 × 10^4^ human melanocytes/cm^2^ into athymic mice and found that mice with the highest density of melanocytes were significantly darker than mice in the other groups. Duval et al. (2014) confirmed that dermal fibroblasts influence the degree of skin pigmentation by measuring quantitative parameters related to skin color, melanin content, and melanocyte numbers in an *in vitro* skin system. Brankov, Prodanovic & Hurley (2016) found that pigmented basal cell carcinomas (BCCs) have a higher mean melanocyte count compared to non-pigmented BCCs, indicating that the pigment is increased not only because of increased melanin, but also because of increased melanocyte counts. The density of melanocytes varies with the body site, with approximately 900 melanocytes per square mm on the back and approximately 1,500 melanocytes per square mm in the genital region (Thingnes et al., 2012). Therefore, for the practical treatment of patients with skin color disorder, the implanted skin should be evaluated for both melanin production and melanocyte count from the donor site for precise color matching. We predict that regulating skin color can be determined by native cell-based melanin production (*M*) and melanocyte numbers in the epidermis (*R*). We found that the *L*^∗^ value was correlated with the value of the multiplicative product of the epidermal melanocyte ratio (*R*) and melanin content (*M*), and these values were much higher than those of the two aforementioned parameters individually, suggesting that considering the epidermal melanocyte ratio and melanin content strengthens the prediction of skin color. Moreover, we performed statistical analysis and found a negative correlation between *M* and *R* (*r* =  − 0.5359, *P* = 0.04). On normalizing *M* to *R* (*M*∕*R*) vs *L*^∗^, a low correlation (*r* = 0.3340, *P* = 0.22) was observed. This result also agreed with the result that the product of *M* and *R* is close to a fixed value.

Although skin pigmentation is known to be regulated by melanocytes, the factors affecting and regulating ethnic skin color are further to be determined (Hoath & Leahy, 2003; Snell & Bischitz, 1963). Based on our previous research, skin color is essentially affected by the ethnicity of the individual, while melanocyte density and differentiation are also influenced by the environmental factors, such as ultraviolet radiation (UVR) and factors secreted by neighboring keratinocytes and fibroblasts (Dai et al., 2018). The melanocortin 1 receptor (MC1R), a G protein-coupled receptor that regulates the quantity and quality of melanin production, is the major determinant of the pigment phenotype of the skin. Three agonists, such as alpha-melanocyte stimulating hormone, adrenocorticotrophic hormone, and proopiomelanocortin, can activate MC1R via the cyclase/cAMP/protein kinase A signaling pathway. Next, the cAMP response element binding protein is phosphorylated, resulting in the transcriptional induction of microphthalmia-associated transcription factor (MITF). MITF is involved in regulating the expression of melanogenic proteins, such as tyrosinase (TYR), tyrosinase-related protein 1, and tyrosinase-related protein 2 to regulate skin color (Gillbro & Olsson, 2011). MITF activity can also be regulated by different transcription factors or mediators secreted by keratinocytes and fibroblasts, such as basic fibroblast growth factor, stem cell factor, endothelin-1, prostaglandins, and leukotrienes. Based on a genome-wide association study, polymorphisms in three genes, including *SLC24A5*, *TYR*, and *SLC45A2*, showed highly significant associations with melanin content in the skin (Stokowski et al., 2007; Wilson et al., 2013). The *SLC24A5* gene encoding the NCKX5 protein, as a potassium-dependent sodium-calcium exchanger, exhibits lower exchange activity, resulting in reduced melanogenesis and lighter skin in individuals (Wilson et al., 2013). These regulatory genes in melanocytes are suggested to differ from race to race or even among individuals and lead to different melanin production capabilities (Han, Choi & Son, 2006). Alaluf et al. (2002a) studied the ethnic variation of melanin content and composition in the human skin from photoprotected and photoexposed areas on human bodies. They found that melanosome size plays a significant role in the variation of different ethnic skin types; African skin had the largest melanosomes followed in turn by Indian, Mexican, Chinese, and European. In addition, the levels of light-colored, alkali-soluble melanin including pheomelanin and DHICA-enriched eumelanin in photoprotected skin areas for European, Chinese, and African skin are estimated as 43.2, 34.4, and 15.1%, respectively (Alaluf et al., 2002a).

In our study, *L*^∗^ value was correlated with melanin content but not epidermal melanocyte ratio. A similar density of pigment-producing melanocytes in the skin (∼1,000/mm^2^) has been found in skin types from different races (Staricco & Pinkus, 1957). However, the skin color of people from the same group may vary because of different living habits, such as the time spent outdoors, diet, or the use of sunscreen, among other factors. Taiwanese people comprise a multi-racial population, including aborigines and immigrants from the mainland. This area was colonized by the Dutch and Japanese in the past. Therefore, the skin color may reflect different racial characteristics showing high variation in skin samples in this study. In fact, there are few more crucial factors affecting final skin color, including the amount of melanin, melanin composition, and melanosome size across skin from a range of ethnicities. In addition, the higher level of melanin production in darker skin was due to the continuously higher level of tyrosinase activity in melanocytes. Taken together, the amount of melanin in the epidermis plays an important role in skin color of individuals from different races, which agrees with our result that melanin content was significantly correlated with *L*^∗^ value of CIELAB color space.

Given that the native melanin-producing capability of a melanocyte depends on genetic factors (Fitzpatrick, Miyamoto & Ishikawa, 1967; Szabo et al., 1969; Wakamatsu et al., 2006), in clinical applications for autogenous pigment cell therapy, the cell-based melanin content is constant in the same individual, and the predicted skin color matches represented as *L*^∗^ value could be detected from the normal skin area adjacent to the hypopigmented lesion site. Thus, a meticulously estimated epidermal melanocyte ratio for the purpose of autogenous pigment cell therapy applications would be attained based on the color formula, which presents as *L*^∗^ = 0.095 × (*M* × *R*) + 55.872 (*M*: melanin concentration per 10^6^ melanocytes; *R*: epidermal melanocyte ratio). Moreover, when culturing various melanocyte ratios in the collagen/PCL scaffolds, the results from *in vitro* experiments confirmed that the use of this skin color formula is feasible. The *L*^∗^ value of L40 and L50 groups was 43.50  ± 1.87 and 50.67 ± 0.55 respectively, very near the predicted value. Therefore, predicting skin color is possible via collecting skin data for establishing the skin color formula. In the clinic, the product of melanin production and melanocyte distribution (M × R) is indicative of the darkness of skin color. The density of melanocytes differs in different parts of the body. For skin autograft, a plastic surgeon often obtains skin grafts from different body sites for implantation at the recipient site. Based on the skin color formula derived in our study, we can more precisely predict skin color by measuring melanin production by the donor skin and adjusting the ratio of melanocytes and keratinocytes to produce a specific color in transplanted skin to treat patients with skin color disorders, such as vitiligo or burn. The cell sheet technique or plasma gel can be used, adjusting the ratio of melanocytes and keratinocytes to prepare transplanted skins suitable for different skin color lesions.

The skin color formula still has some limitations that should be addressed before its clinical application. Swope et al. found that cutaneous pigments would be present as a function of melanocyte density and time after grafting due to the depletion of human melanocytes in CSS in an animal model (Swope et al., 1997; Swope, Supp & Boyce, 2002). Furthermore, skin color is suggested to be determined by several other factors including the total quantity of melanin, the proportion between the brown–black eumelanin and the yellow–red pheomelanin, and its distribution involved in the epidermis (Naysmith et al., 2004). The log_e_ values of eumelanin/pheomelanin ratio were inversely related to the color variables *b*^∗^ (yellow–blue) (*R*^2^ = 0.51, *P* < 0.001), *a*^∗^(red–green)(*R*^2^ = 0.47, *P* < 0.001), and to a lesser extent *L*^∗^ values (*R*^2^ = 0.22, *P* < 0.001) (Naysmith et al., 2004). On the other hand, three of cell types including melanocytes, keratinocytes, and fibroblasts actively participate in regulating skin pigmentation through secreted factors and their receptors, and their interactions may determine skin pigmentation (Yin et al., 2014). The other chromophores present in the skin include oxyhemoglobin, reduced hemoglobin, and carotene, which may influence the true skin color. Skin blood flow increases with increases in hemoglobin (Tsuchida, Fukuda & Kamata, 1991), and *a*^∗^ values correlate linearly well with hemoglobin levels. The *a*^∗^ value indicates the redness of the skin color and is mainly influenced by the degree of vascularization and the stretching of the skin over surrounding tissues. Therefore, all three CIE color-space parameters: *L*^∗^ value, *a*^∗^ value, and *b*^∗^ value should be discussed in the future for perfect match of the skin color used in clinical application.

## Conclusions

A formula for estimating skin color based on CIELAB *L*^∗^ value, epidermal melanocyte ratio, and melanin concentration was generated from people of Asian descent. This skin color formula may serve as a useful methodology for determining distinct pigment cell concentrations for cell therapy or for developing a pigmented skin tissue engineering model for transplantation and pharmaceutical screening applications *in vitro*.

##  Supplemental Information

10.7717/peerj.4815/supp-1 Supplemental Information 1 Analysis of skin pigmentation

10.7717/peerj.4815/supp-2 Supplemental Information 2 Measurement of *L* value in tissue engineering skin

## Additional Information and Declarations

### Data Availability

The following information was supplied regarding data availability: Dai, Niann-Tzyy; Huang, Wen-Shyan; Wang, Yi-Wen; Hung, Kun-Che; Fu, Keng-Yen; Dai, Lien-Guo; Liou, Nien-Hsien; Liu, Jiang-Chuan (2018): Analysis of skin pigmentation. figshare. Dataset. https://doi.org/10.6084/m9.figshare.5797755.v1. Dai, Niann-Tzyy; Huang, Wen-Shyan; Wang, Yi-Wen; Hung, Kun-Che; Hsieh, Pai-Shan; Fu, Keng-Yen; Dai, Lien-Guo; Liou, Nien-Hsien; Ma, Kuo-Hsing; Liu, Jiang-Chuan (2018): Measurement of L value in tissue engineering skin. figshare. Dataset. https://doi.org/10.6084/m9.figshare.5797749.v2.
